# Screening and Brief Intervention in the Emergency Department

**Published:** 2004

**Authors:** Gail D’Onofrio, Linda C. Degutis

**Affiliations:** Gail D’Onofrio, M.D., M.S., is an associate professor in the Department of Surgery, Section of Emergency Medicine, and interim chief of the Section of Emergency Medicine, Yale University School of Medicine, New Haven, Connecticut. Linda C. Degutis, Dr. P.H., is an associate professor in the Department of Surgery, Section of Emergency Medicine, and research director of the Section of Emergency Medicine, Yale University School of Medicine, New Haven, Connecticut

**Keywords:** hazardous AOD (alcohol and other drug) use, harmful AOD use, alcohol abuse, emergency room, trauma center, drinking and driving, identification and screening, intervention (persuasion to treatment), brief intervention, counseling, motivational interviewing, barriers to treatment, literature review

## Abstract

Many patients visiting hospital emergency departments (EDs) or admitted to trauma centers have alcohol problems. Therefore, it is plausible that all ED and trauma patients should be screened for unhealthy alcohol use so that optimal care can be provided and treatment initiated, if necessary, for these patients. In addition, brief interventions offered directly in the ED or trauma unit could be useful for many patients. Some studies have found such interventions to be feasible and effective in this setting. However, all efforts in this regard must take into consideration the specific challenges associated with screening and intervention in EDs, such as time constraints, ethical and legal issues, and concerns regarding insurance coverage. Innovative approaches to screening may address at least some of these problems, although more research is needed to determine how screening can be better incorporated and implemented in the ED setting.

Many patients visiting hospital emergency departments (EDs) exhibit unhealthy alcohol use (Saitz 2005), which encompasses patterns of alcohol consumption that put the drinker at risk for adverse consequences (known as at-risk drinking), have led to alcohol-related problems but do not meet the criteria for an alcohol use disorder (known as problem drinking), or meet the criteria for an alcohol use disorder (i.e., alcohol abuse or alcohol dependence). (For more information, see the textbox.) As a result, ED practitioners routinely care for patients with adverse health effects associated with alcohol consumption. This article examines the prevalence of alcohol-related health problems among ED patients, reviews studies evaluating the effectiveness and feasibility of brief interventions in the ED setting, and points out particular challenges associated with screening and brief interventions in this setting, including ethical and legal barriers to screening and intervention. In addition, innovative approaches to screening and intervention in the ED are presented, and issues that need to be addressed in future studies are discussed.

## Prevalence of Unhealthy Alcohol Use in ED Patients

A substantial portion of the estimated 110 million ED visits in the United States each year are related to unhealthy alcohol use. As many as 24 to 31 percent of all patients who are treated in an ED and as many as 50 percent of severely injured trauma patients (i.e., patients who require hospital admission, usually to an intensive care unit, for treatment of acute injuries) have positive results when screened for alcohol problems (D’Onofrio and Degutis 2002). In addition, patients treated in EDs are 1.5 to 3 times more likely than those treated at primary care clinics to report heavy drinking, adverse consequences of drinking (e.g., alcohol-related injuries, illnesses, and legal or social problems), or having ever been treated for an alcohol problem (Cherpitel 1999).

The prevalence of alcohol use disorders in ED patients was confirmed by a study conducted in seven representative EDs across Tennessee, in which patients were assessed to determine their need for alcohol and other drug (AOD) treatment (Rockett et al. 2003). The researchers reported that, based on the assessment, as many as 27 percent of ED patients needed AOD treatment services; however, in only 1 percent of the cases did the ED physicians document a diagnosis of AOD abuse in the patients’ medical files. Moreover, less than 10 percent of the patients determined to be in need of AOD treatment actually received any. Patients were more likely to need AOD treatment services if they were insured by Medicaid or Medicare, had come to the ED 2 or more hours after the onset of the illness or injury (i.e., had delayed the ED visit), or had visited the ED more than once. Interestingly, no difference in treatment need existed between patients visiting the ED because of an injury (e.g., from an alcohol-related car crash or fall) and patients visiting the ED for other illnesses. Finally, treatment need was inversely associated with age—that is, younger patients were more likely to need AOD treatment than older patients.

Several factors contribute to the fact that younger ED patients may be more likely to have alcohol-related problems that indicate a need for treatment. First, younger people are usually healthy and are more likely than older people to be uninsured and to use the ED as their usual source of care (McCarthy et al. 2002).^1^ Second, young adults have the highest prevalence of binge^2^ and hazardous drinking in the United States (SAMHSA 2003*a*), which can easily escalate to drinking patterns that require intervention.

Third, particularly in younger people, these drinking patterns often occur in conjunction with driving. According to the 2001 National Household Survey on Drug Abuse (SAMHSA 2003*b*), 3 million people ages 16 to 20 had driven under the influence of alcohol at least once in the past year, including 600,000 people ages 16 or 17. Motor vehicle crashes are the number one cause of death in people ages 1 to 35, and the eighth leading cause of death overall (CDC 2004). In 2003 (the most recent data available), there were approximately 43,000 motor vehicle traffic fatalities in the United States, according to the National Highway and Traffic Safety Administration (NHTSA), of which an estimated 18,000 (40 percent) were related to the use or abuse of alcohol (NHTSA 2005). Consequently, NHTSA has made prevention of impaired driving a major initiative and is working to encourage health care practitioners (including those in EDs) to provide screening and brief intervention services. This initiative supports the Institute of Medicine’s (1990) landmark report on broadening the base of AOD abuse treatment, which recommends that patients in all medical settings should be screened for problems along the entire spectrum of unhealthy alcohol use and that they be offered brief intervention and referral to treatment services.

**Definitions of Unhealthy Alcohol Use**

The term “unhealthy alcohol use” refers to a spectrum of disorders ranging from at-risk drinking to alcohol dependence. At-risk or hazardous drinking implies that the person is drinking over the recommended limits and is therefore vulnerable to illness, injury, or social/legal problems. These recommended consumption limits are, for men, 2 standard drinks per drinking occasion or 14 standard drinks per week, and, for women and people age 65 and over, 1 standard drink per drinking occasion or 7 drinks per week. A standard drink is defined as 12 grams of pure alcohol, the amount contained in approximately 12 oz of beer, 5 oz of wine, or 1.5 oz of distilled spirits.

Once a person experiences an alcohol-related harmful event—an injury, illness, or social/legal problem such as poor grades, an argument with parents, or a driving violation—he or she is classified as a harmful drinker.

The far end of the spectrum includes alcohol abuse and alcohol dependence as defined by the diagnostic criteria that have been established in the American Psychiatric Association’s *Diagnostic and Statistical Manual of Mental Disorders, Fourth Edition* (DSM–IV).

## Effectiveness of Brief Interventions in the ED Setting

ED practitioners are chronically pressed for time, and resources often are limited in this setting. Therefore, if ED practitioners are to be encouraged to screen their patients for alcohol problems and offer brief intervention if necessary, particularly under the time constraints they are facing, they first must be convinced of the usefulness of these measures. Brief interventions are short counseling sessions, ranging from 5 to 60 minutes, performed by nonaddiction specialists. Including the concepts of motivational interviewing (MI) may enhance the success of the intervention in changing patients’ behavior. The principles of MI, developed by Miller and Rollnick (1991), are encapsulated in the FRAMES acronym (feedback, responsibility, advice, menu or choice, empathy, and self-efficacy). The goal of the brief intervention is to assist patients who exhibit less severe patterns of unhealthy alcohol use (i.e., at-risk drinking and problem drinking) to reduce their alcohol consumption to low-risk levels, thereby reducing the risk of illness or injury. For those patients who are alcohol dependent, the goal may be abstinence and/or acceptance of a referral to a specialized treatment program.

Chafetz and colleagues (1962) published the first report on what could be considered a brief intervention in the ED (i.e., a referral to a specialized alcoholism treatment clinic as well as information on strategies to assist with social issues, etc.). Their study demonstrated that the intervention could motivate alcohol-dependent patients to initiate alcoholism treatment. These investigators reported that 65 percent of patients with alcohol dependence who received the intervention and a direct referral from the ED to an alcoholism treatment clinic kept their initial appointment at the clinic, compared with 5.4 percent of the control group, who received just a referral.

Although a number of studies have subsequently evaluated the effectiveness of brief intervention in the ED, these are difficult to compare because they often used restricted populations (e.g., only young adults), involved interventions of varying lengths, and had methodological limitations (D’Onofrio and Degutis 2002). To date, only four studies have analyzed the effectiveness of brief interventions in ED patients according to stringent scientific standards (i.e., by including control groups and randomly assigning participants to an intervention or control group). All four studies, which are described in the following sections, included only injured patients. (For a summary of the characteristics and findings of these studies, see the table.)

### Study Conducted by Monti and Colleagues

Monti and colleagues (1999) compared the effectiveness of “standard care” with that of a brief motivational interview in reducing alcohol-related consequences and alcohol use among ED patients ages 18 and 19. Standard care was described as “consistent with general practice for treating alcohol-involved teens in an urgent care setting” and included a handout on avoiding drinking and driving as well as a list of local treatment agencies. The 94 participants were recruited for the study because they had positive blood alcohol concentrations (BACs) or an alcohol-related injury (i.e., reported drinking prior to the injury that required treatment). Followup assessments were conducted by phone after 3 months and through face-to-face interviews after 6 months. This study found that both groups of patients decreased their alcohol consumption, but that patients who participated in the MI showed significantly greater improvements in the following alcohol-related variables:

- They reported a significantly lower incidence of drinking and driving (62 percent, versus 85 percent in the control group, which received standard care).
- They were less likely to have been cited for a moving violation during the followup period (3 percent versus 23 percent).
- They were less likely to have sustained an alcohol-related injury during the followup period (21 percent versus 50 percent).
- They reported fewer alcohol-related social and legal problems in the 6 months following treatment, such as problems with dates, friends, parents, school, or the police.

### Study Conducted by Longabaugh and Colleagues

Longabaugh and colleagues (2001) evaluated the effects of a brief motivational intervention in injured drinkers age 18 or older who visited the ED of an urban teaching hospital. Patients were eligible for the study if they screened positive for hazardous or harmful drinking (i.e., had breath alcohol concentrations greater than 0.003 mg/dL, reported having ingested alcohol in the 6 hours before the injury, or scored positive on the AUDIT screening test^3^). Participants were randomly assigned to one of three groups:

- Standard care (SC) (*N* = 188).
- A brief intervention (BI) consisting of a 40- to 60-minute session provided by non-ED staff (i.e., a social worker or graduate student) (*N* = 182).
- BI with a booster (BIB) that entailed a scheduled return visit 7 to 10 days after the initial BI (*N* = 169).

At 1 year after the intervention,^4^ participants in all three groups reported having reduced their days of heavy drinking, similar to the findings of the study by Monti and colleagues (1999). Moreover, the BIB group reported significantly fewer alcohol-related negative consequences (e.g., hangovers and lost work time) and alcohol-related injuries than did the SC group. However, the average number of injuries the participants had sustained in the year preceding the ED visit was low (i.e., an average of 1.6 injuries), as was the incidence of new injuries. Therefore, it is difficult to demonstrate significant changes, which limits the interpretation of the findings. Nevertheless, the investigators concluded that a booster added to a brief intervention with injured ED patients who engage in hazardous and harmful drinking may be helpful in reducing negative consequences and alcohol-related injuries.

### Study Conducted by Spirito and Colleagues

Spirito and colleagues (2004) studied adolescents ages 13 to 17 who were treated in an ED for an alcohol-related event. The adolescents were eligible to participate in the study if they had evidence of alcohol in their blood, breath, or saliva (*N* = 142), or if they reported drinking alcohol in the 6 hours before the injury that required treatment in the ED (*N* = 10). The participants underwent a battery of assessments that took an average of 45 minutes to complete. They reported their drinking behavior over the past 12 months and completed the Adolescent Drinking Questionnaire (which assesses behavior over the past 3 months), the Young Adult Drinking and Driving Questionnaire, and the Adolescent Injury Checklist. Furthermore, at the beginning of the study the investigators administered the Adolescent Drinking Inventory (ADI) to identify adolescents with potential alcohol problems warranting a treatment referral and for use in the personal feedback component of the intervention condition. The ADI is a 24-item measure of severity of alcohol involvement, with a score > 15 indicating that referral for alcohol problems is needed. Participants then were randomly assigned to receive standard care or a brief motivational intervention.

Researchers interviewed the adolescents by phone after 3 months and contacted them in person after 6 and 12 months. The investigators found that adolescents in both groups drank less alcohol during the 12-month followup period. However, adolescents in the MI group with a baseline ADI score indicating problematic alcohol use improved significantly in two outcomes, average number of drinking days per month (frequency) and frequency of high-volume drinking (bingeing). Based on these findings, the investigators recommend that adolescents who are treated in the ED for an alcohol-related injury should be screened for preexisting alcohol problems and should receive a brief intervention if the screen is positive.

### Limitations of These Studies

Several methodological issues may have influenced the results and limited the generalizability of the three studies described so far. First, all three had high refusal rates—that is, as many as 47 percent of patients in the studies refused to participate. Refusal rates of this magnitude can introduce significant bias (e.g., only patients who have less severe problems or are more willing to change their drinking behavior may agree to participate). Refusal rates in studies involving adolescents may be particularly high because parents may need to give consent for their children to participate, and adolescents may not want their parents (or others) to know about their alcohol consumption for fear of getting in trouble because of underage drinking.

Second, the SC conditions in the studies did not truly represent the standard of care. Other studies have demonstrated that emergency practitioners rarely screen their patients for alcohol problems or provide any intervention (D’Onofrio and Degutis 2002). In the three studies, however, patients in the SC groups received at least brief advice and a handout on avoiding drinking and driving—minimal interventions that nonetheless go beyond the standard of care commonly seen in ED settings.

**Table:** Comparison of Four Clinical Studies Evaluating the Effectiveness of Brief Interventions in Emergency Departments and Inpatient Trauma Units*

Study	Study Design and Setting	Patient Population and Admission Criteria	Intervention	Followup Rate	Outcome	Effect
Monti et al. 1999	Design: RCT Setting: ED	94 patients ages 18–19, treated at an ED after an alcohol-related event Positive BAC or Report of drinking prior to the event that precipitated treatment	Standard care One 35- to 40- minute BI (motivational interview) Interventions performed by 12 experienced research assistants (bachelor’s and master’s level) No followup sessions	3 months (phone): 93% 6 months (in person): 89%	Decrease in alcohol consumption in both groups Greater reduction in alcohol-related injuries during the followup period in the BI group Greater reduction in other alcohol-related problems (e.g., drinking and driving, social and legal problems) in the BI group	Positive effect with the BI
Gentilello et al. 1999	Design: RCT Setting: Inpatient Trauma Center	762 patients ages ≥ 18 admitted to a trauma center BAC ≥100 mg/dL or SMAST score ≥3 or BAC 1–99 mg/dL and SMAST score of 1 or 2 or BAC 1–99 mg/dL and elevated GGT or SMAST score of 1 or 2 and elevated GGT	Standard care One 30-minute BI (motivational interview) Interventions performed by one Ph.D.-level psychologist Followup letter sent after 1 month	6 months: 75% 12 months: 54%	Greater reduction in alcohol-related injuries during the followup period in the BI group Greater decrease in alcohol consumption in the BI group Greater reduction in ED visits and hospitalizations in the BI group	Positive effect with the BI
Longabaugh et al. 2001	Design: RCT Setting: ED	539 patients ages ≥ 18 with evidence of harmful or hazardous drinking, whose injury did not require hospitalization BAC≥ 0.003 mg/dL or Report of alcohol use 6 hours prior to injury or AUDIT score ≥8	Standard care One 40- to 60- minute BI One 40- to 60- minute BI followed by scheduled return visit (booster) 7–10 days later (BIB) Interventions performed by 8 clinically experienced research assistants (Ph.D., master’s, or bachelor’s level)	1 year (phone, mail, in person): 83%	Greater reduction in alcohol-related injuries during the followup period in the BIB group Decreases in alcohol consumption in all groups Greater reduction in alcohol-related negative consequences in the BIB group	Positive effect with the BIB
Spirito et al. 2004	Design: RCT Setting: ED in an urban level 1 trauma center	Adolescents treated in an ED after an alcohol-related event Positive for alcohol in breath, saliva, or blood or Self-reported alcohol use 6 hours prior to injury Note: 47% of adolescents asked to participate refused	Standard care (5 minutes) One 35- to 45- minute BI (motivational interview) Interventions performed by 12 clinically experienced research assistants (bachelor’s and master’s level) No followup sessions	3 months (phone): 93.4% 6 months (in person): 89.5% 12 months (in person): 89.5%	Greater reduction in frequency of drinking and binge drinking for patients with preexisting problematic alcohol use in the BI group	Positive effect with the BI for problem drinkers

* RCT = randomized controlled trial, ED = emergency department, BAC = blood alcohol concentration, BI = brief intervention, BIB = brief intervention with booster, SMAST = Short Michigan Alcohol Screening Test, AUDIT = Alcohol Use Disorders Identification Test, GGT = gamma glutamyltransferase.

Third, the screening and assessment of the participants may have acted like an intervention, as indicated by the fact that participants in all study groups decreased their alcohol use. Assessment questionnaires that take 30–45 minutes to complete may have an impact similar to that of brief interventions of similar duration. Furthermore, questionnaires such as the Adolescent Injury Checklist, Adolescent Drinking Inventory, and Drinking Inventory of Consequences emphasize the consequences of alcohol misuse and in themselves may provide feedback that motivates people to think about their behavior; such feedback is one of the key components of the intervention being tested.

Fourth, to detect statistically significant differences between control and intervention groups, adequate numbers of patients must participate and be randomly distributed to the different groups. The three studies discussed, however, did not report conducting an analysis to determine if their sample size was sufficient to detect differences (i.e., a power analysis). This makes interpretation of the studies’ findings difficult, particularly because none of the studies found a significant difference between the intervention and control groups with respect to alcohol consumption. Without a power analysis, it is difficult to determine whether this lack of differences is genuine or just results from inadequate sample sizes. Power analyses also should be conducted for analyses of the study data on negative consequences of alcohol consumption (e.g., drinking and driving or number of alcohol-related injuries). Such power analyses may be challenging, however, because the prevalences of such consequences may be low at baseline.

### Study Conducted by Gentilello and Colleagues

The fourth study, conducted by Gentilello and colleagues (1999), avoided at least some of the methodological limitations of the other three studies. However, this study included only hospitalized trauma patients who were found to exhibit unhealthy alcohol use based on screening and/or testing.^5^ It did not include patients who were only treated in the ED and released. The participating patients represented the full spectrum of unhealthy alcohol use. The patients then were randomly assigned to either the control group or the intervention group, which received a single 30-minute motivational interview conducted by a doctoral-level psychologist in the inpatient setting.

Followup with the participants was conducted after 6 and 12 months. Although only 54 percent of participants were available for the followup at 12 months, the investigators found that those in the intervention group decreased their weekly alcohol consumption significantly more (by 21.8 drinks) than the control group (by 6.7 drinks). The decrease was greatest in patients with mild to moderate alcohol problems at the beginning of the study. Furthermore, the beneficial effects of the intervention appeared to be persistent, because after a 3-year followup period, the investigators found a 47-percent reduction in injuries requiring an ED visit or readmission to the trauma service in the intervention group (Gentilello et al. 1999). Because of the low followup rate, however, it is not possible to generalize the findings of this study, as one cannot determine whether the patients who were not followed up also decreased their alcohol consumption.

The difference between this and the other three studies, which may improve the generalizability of these results, is the inclusion of a more credible control group. The control group in this study received minimal screening and assessment, which were less likely to have acted as an intervention and to have confounded the results. Also, in contrast to the other studies, this investigation included patients who had sustained injuries significant enough to warrant admission to the hospital, which in itself may lead to a so-called teachable moment and may contribute to the patients’ motivation to change their behavior. Limitations of the study included a relatively high refusal rate (34 percent of eligible patients did not participate) and a relatively low followup rate. Moreover, patients who are hospitalized because of injuries make up only a small proportion of the patients with alcohol problems who present to the ED for treatment of injuries, and the findings may therefore not apply to all patients with alcohol problems.

### Applicability of Study Findings in Everyday ED Practice

All four studies have suggested that providing some form of brief intervention to ED patients whose injuries are alcohol related may decrease their alcohol consumption and alcohol-related negative consequences. The specific message that should be delivered to the patients, however, is not so clear, because the standard care groups—which received some brief advice, information, or assessments containing motivational statements—also experienced positive outcomes. Also, because the interventions in these studies were implemented by research staff, who are not typically available in most EDs (i.e., social workers, graduate students, or doctoral-level psychologists), it is unclear how the findings can be translated into the real-world ED setting. Other research on the feasibility of screening and brief interventions in the ED setting can shed additional light on this question.

Degutis (1998) demonstrated that screening with tools such as quantity/frequency questions and the four-item CAGE questionnaire was feasible in a real-world ED setting. (For information on the CAGE and other commonly used screening instruments, see the sidebar “Screening Tests,” on page 78 of this issue.) Similarly, Hungerford and colleagues (2003), using a study population of young adults ages 18 to 39, reported that screening and intervention could be integrated in the ED setting. In this study, research staff screened a convenience sample^6^ of ED patients who were waiting for treatment. The investigators found that 87 percent of the young adult drinkers consented to screening. Of these, 43 percent screened positive for alcohol problems on the AUDIT,^7^ and of those with positive screens, 94 percent received counseling. The high prevalence of alcohol problems and the broad acceptance of screening and brief intervention in this sample indicated that even though the study used research staff, who are not present in real EDs, screening is feasible in this setting, and the ED is a promising venue for screening and brief intervention.

A survey of emergency practitioners (D’Onofrio et al. 2004) found that these clinicians considered performing a brief intervention for harmful and hazardous drinkers feasible and acceptable in their everyday practice. Other investigators demonstrated that emergency medicine residents who received training in screening and brief intervention in a skills-based workshop increased their knowledge and practice of these procedures (D’Onofrio et al. 2002). Fifty-eight percent of medical records of patients treated by trained residents contained evidence of screening and intervention, compared with 17 percent of records of patients treated by a control group of similar residents who did not receive training.

## Challenges Associated With Screening and Intervention in the ED Setting

Many barriers to screening, brief intervention, and referral have been identified in the ED setting. This environment is always chaotic, and time is precious. Lack of confidence on the part of the emergency practitioners regarding their ability to screen patients effectively, scarcity of role models who are performing screening, and inadequate resources often are cited as reasons why practitioners fail to screen and intervene. In addition, ethical matters and insurance constraints may present obstacles.

### Time Constraints

To identify potential barriers more accurately, Graham and colleagues (2000) surveyed 569 members of the Michigan College of Emergency Physicians about their attitudes toward using interventions with ED patients who have alcohol problems. Of the 257 members who responded (46 percent of those surveyed), 75 percent agreed that alcohol abuse and dependence are treatable illnesses, and only 15 percent stated they would not support ED interventions. Both supporters and nonsupporters thought that lack of time was a major obstacle to screening and intervention. Consequently, the study’s authors suggested that existing interventions be adapted to the time limitations of the ED setting. One type of brief intervention that can be performed in less than 10 minutes already has been developed and tested specifically for emergency practitioners (D’Onofrio et al. 1998).

### Ethical and Legal Issues

Several ethical and legal issues frequently arise in the discussion of screening and brief intervention for alcohol problems in the ED as well as in other health care settings. One of the most important issues is the relationship between patient and practitioner, and the practitioner’s responsibility to identify behaviors such as unhealthy alcohol use that can result in health problems. Identifying such behaviors may create a dilemma for practitioners who are uncertain about how to intervene and where to refer these patients without compromising the patient–physician relationship. Nevertheless, practitioners have an obligation to identify health risks associated with alcohol use and to treat the alcohol use problems themselves or refer the patients to the appropriate resources, just as they would do for any other chronic disease, such as hypertension or diabetes (McLellan et al. 2000). To illustrate, screening for alcohol problems often is compared with screening for tetanus immunization. Every injured ED patient is asked whether he or she has been vaccinated against tetanus and is offered treatment, no matter how busy the practitioner is. Even though most practitioners have never even seen a case of tetanus, they allocate time for this screening. Therefore, considering the high number of ED patients suffering from alcohol-related injuries and other adverse consequences, it is difficult to understand why practitioners often ignore this health problem, which they typically encounter during each shift.

In order to make the process of screening, intervention, and referral as easy as possible and thereby promote its use, the American College of Emergency Physicians (2004) has developed an *Alcohol Screening and Brief Intervention Resource Kit* that is available via the Internet. This toolbox contains an explanation of brief interventions, samples of patient handouts, and information about how to develop resource lists for individual communities.

### Insurance Coverage

Some health practitioners also are reluctant to screen their patients for alcohol use because they are concerned that if they identify an alcohol problem, the patients’ health insurance carrier may deny reimbursement for the ED visit. This concern is especially prevalent in ED and trauma care settings, because many States have insurance regulations allowing insurers to exclude coverage for a loss sustained because the insured was under the influence of alcohol and other drugs (Rivara et al. 2000). These exclusions are based on a widely adopted model called the Uniform Accident and Sickness Policy Provision Law. (For more information on the denial of health care coverage for ED visits for AOD-related problems, see the accompanying sidebar by Chezem.) Only two States (Iowa and South Dakota) specifically prohibit insurers from denying coverage in this situation.

The arguments related to denial of insurance coverage, however, do not seem justifiable in the context of screening because screening differs from testing (e.g., determination of blood alcohol levels). To deny reimbursement, the insurer must demonstrate that AOD use to some degree caused the insured’s injury or other problem. This demonstration generally is based on a diagnostic or laboratory test documenting a specific AOD concentration in the patient’s blood, which is linked to the reason for the ED visit or hospitalization. Screening using structured questionnaires, in contrast, identifies an existing problem without linking it directly to the patient’s current visit and therefore provides an opportunity to intervene. Thus, it probably would be difficult for an insurer to connect the fact that a person drinks more than recommended by national drinking guidelines to an ED visit for a wrist fracture that occurred when the patient slipped on ice going to work. Consistent with this assumption, all legal cases related to the denial of coverage have been based on testing that was done at the time of an incident but were not based on screening for a preexisting alcohol problem.

The larger issue of substance abuse treatment parity—that is, regulations requiring that alcohol and other drug abuse treatment be reimbursed at the same level as treatment for other diseases—still remains. A Federal law requiring insurance coverage parity for AOD abuse treatment was first introduced in both Houses of Congress in 1997 but has yet to pass. A few States have laws requiring parity, but these laws only apply to insurance plans that are regulated by the States and do not include Federally regulated plans (e.g., the Federal Employees Health Benefit Plan) that are governed by Employment Retirement Income Security Act (ERISA) statutes.

## Innovative Approaches to Screening in the ED

Several studies have reported innovative methods for screening and intervention in the ED. Rhodes and colleagues (2001) described the use of a computer-based approach to screening and general health promotion in the ED. (This approach was not specific to AOD-related problems but addressed a variety of health issues.) In this study, 542 adult ED patients with nonurgent conditions (89 percent of those approached) were assigned either to the computer intervention—a self-administered computer survey generating individualized health information—or to usual care (i.e., no intervention). In the intervention group, 85 percent of participants reported one or more behavioral risk factors for AOD problems, including problem drinking (19 percent), or driving within 4 hours of having two or more drinks (11 percent). In addition, 95 percent of patients in the intervention group requested further health information. One week after the ED visit, 62 percent of the intervention group remembered receiving advice on what they could do to improve their health. The investigators concluded that computer technology may help physicians use the patients’ waiting time for health promotion and to target patients at risk for various health problems.

Gregor and colleagues (2003) examined the feasibility of using an interactive computer program in the ED to prevent alcohol misuse among adolescents, enrolling patients ages 14 to 18 who visited the ED within 24 hours of an acute injury. Of the participants, 71 percent reported ever drinking alcohol, and about 63 percent reported recent alcohol use. The program consisted of an interactive house party with audio. Each participant chose a “party pal” from a group of five teenaged cartoon characters and was exposed to various scenarios depicting important concepts regarding alcohol misuse. Of the recent drinkers participating in the study, 74 percent reported that the program made them rethink their alcohol use, 94 percent liked the program, and only about 5 percent required assistance with it. An ongoing study currently is evaluating the effectiveness of the program in reducing participants’ alcohol-related problem behaviors.

Another approach, being studied at Boston Medical Center and Yale–New Haven Hospital, is the Project ASSERT model, which uses Health Promotion Advocates (HPAs) or community outreach workers to screen, intervene, and refer patients with alcohol problems. Bernstein and colleagues (1996) evaluated the effectiveness of this program in 245 participants, most of whom had alcohol dependence. After 90 days, these participants reported a 56-percent reduction in alcohol use and a 64-percent reduction in binge drinking. In addition, more than 50 percent of participants had received a treatment referral. In another evaluation of Project ASSERT, 10,572 patients were screened and evaluated, and 1,343 patients were referred to specialized AOD treatment programs over a 2-year period (D’Onofrio and Degutis 2003). HPAs were subsequently able to contact 811 of the referred patients, of which 711 (88 percent) had enrolled in a treatment program. The results suggest that this model of screening and direct linkage to treatment is feasible. Moreover, the program is likely to be sustainable, because Project ASSERT is funded by HPA consultation fees that are included in the hospitals’ billing processes.

## Future Directions

The studies discussed in this article have suggested that screening and brief intervention in the ED can be feasible and effective, but more research is necessary to determine optimal treatment approaches and their implementation in the ED setting. Such research should

- Include both injured and noninjured patients to reflect the heterogeneous patient population seen in EDs.
- Include sample sizes adequate to detect differences between intervention and control groups on key outcome variables such as alcohol consumption and other negative consequences.
- Include control groups exposed to minimal assessments so as to minimize the potential influence of the assessment on the patients and thus compare interventions with true “usual care.”
- Evaluate specific components of interventions (e.g., motivational enhancement or simple feedback and advice) to determine which components are most effective in the ED setting. Such analyses could clarify whether intervention effectiveness depends on the patients’ readiness to change and/or the severity of their injury or illness, whether “boosters” are beneficial and cost-effective, whether followup and boosters should be conducted by phone or in person, or whether self-reported outcomes should be obtained by phone, in person, or by interactive voice response.
- Explore how screening and intervention can best be incorporated into clinical practice, whether existing ED staff (e.g., nurses, physicians, physician extenders) can be used at least to some extent, and what other alternatives (e.g., peer educators, interactive computerized methods, or volunteers) are available.
- Establish the cost–benefit ratios of various interventions, determine the most effective method of recovering costs, and explore how practitioners can be adequately reimbursed for any counseling they provide.
- Investigate how to overcome obstacles to screening and intervention, such as difficulties with insurance coverage and unwillingness or inability of practitioners to perform these measures.
- Address how screening and brief intervention skills can be incorporated in the practitioners’ initial professional education.
- Assess screening and intervention for culturally diverse groups and non-English-speaking patients, who represent a significant proportion of ED patients but have not been a focus of existing studies.

## Conclusions

ED visits provide health care practitioners with an important opportunity to screen their patients for alcohol problems and, if necessary, to initiate brief intervention. Research has demonstrated that screening and brief interventions are feasible and effective in the ED setting. However, to be successful in changing physicians’ daily practices and decreasing the harmful consequences of alcohol misuse, clinicians, researchers, and policy-makers still must address a variety of research questions and policy issues.

## Footnotes

1 The various studies define “older” and “younger” differently. Most commonly, “younger people” are those age 25 and younger.

2 Binge drinking as it is used here is defined as consumption of five or more drinks on one drinking occasion; it also is called heavy episodic drinking.

3 The Alcohol Use Disorders Identification Test (AUDIT) is a commonly used screening tool to detect hazardous or harmful drinking. A score of 8 points or more generally indicates that a patient is likely to meet the criteria for hazardous or harmful drinking.

4 For the followup, it was possible to contact 84 percent of the original participants—39 percent in person and 61 percent by mail or phone.

5 Screening involved completion of a questionnaire, whereas testing refers to analyses of alcohol levels in the blood, saliva, or breath.

6 The term “convenience sample” means that the investigators picked potential participants at will, rather than just using consecutive patients or another preset selection scheme. This process may introduce some bias into the sample.

7 In this study, a score of > 6 points was considered a positive screen.
